# The Effects of Mechanical Stress on the Growth, Differentiation, and Paracrine Factor Production of Cardiac Stem Cells

**DOI:** 10.1371/journal.pone.0028890

**Published:** 2011-12-28

**Authors:** Hiroshi Kurazumi, Masayuki Kubo, Mako Ohshima, Yumi Yamamoto, Yoshihiro Takemoto, Ryo Suzuki, Shigeru Ikenaga, Akihito Mikamo, Koichi Udo, Kimikazu Hamano, Tao-Sheng Li

**Affiliations:** 1 Department of Surgery and Clinical Science, Yamaguchi University Graduate School of Medicine, Ube, Yamaguchi, Japan; 2 Institute for Biomedical Research and Education, Yamaguchi University Science Research Center, Ube, Yamaguchi, Japan; 3 Department of Stem Cell Biology, Nagasaki University Graduate School of Biomedical Science, Nagasaki, Japan; Medical College of Georgia, United States of America

## Abstract

Stem cell therapies have been clinically employed to repair the injured heart, and cardiac stem cells are thought to be one of the most potent stem cell candidates. The beating heart is characterized by dynamic mechanical stresses, which may have a significant impact on stem cell therapy. The purpose of this study is to investigate how mechanical stress affects the growth and differentiation of cardiac stem cells and their release of paracrine factors. In this study, human cardiac stem cells were seeded in a silicon chamber and mechanical stress was then induced by cyclic stretch stimulation (60 cycles/min with 120% elongation). Cells grown in non-stretched silicon chambers were used as controls. Our result revealed that mechanical stretching significantly reduced the total number of surviving cells, decreased Ki-67-positive cells, and increased TUNEL-positive cells in the stretched group 24 hrs after stretching, as compared to the control group. Interestingly, mechanical stretching significantly increased the release of the inflammatory cytokines IL-6 and IL-1β as well as the angiogenic growth factors VEGF and bFGF from the cells in 12 hrs. Furthermore, mechanical stretching significantly reduced the percentage of c-kit-positive stem cells, but increased the expressions of cardiac troponin-I and smooth muscle actin in cells 3 days after stretching. Using a traditional stretching model, we demonstrated that mechanical stress suppressed the growth and proliferation of cardiac stem cells, enhanced their release of inflammatory cytokines and angiogenic factors, and improved their myogenic differentiation. The development of this in vitro approach may help elucidate the complex mechanisms of stem cell therapy for heart failure.

## Introduction

In the past decade, many studies have provided evidence of the potential for cardiac tissue self-regeneration, even in adult mammals and human beings [1]–[5]. This evidence includes a reservoir of cardiac-specific stem cells or progenitor cells found in adult hearts and the increased proliferative activity of cardiomyocytes in failing hearts [1]–[5]. However, the obvious self-regeneration of infarcted hearts was almost never clinically observed [6]. It is possible that the strong dynamic mechanical stresses and an unfavorable environment in the injured heart induce a loss of myocytes that exceeds the heart's weak regenerative ability. Therefore, the implantation of exogenous stem cells is still considering as a promising therapy for heart failure, although the mechanism on stem cell therapy have been recently demonstrated to largely depend on paracrine effects rather than direct regeneration of new functional myocardium [7]–[9].

Many clinical trials have been conducted using the delivery of autologous stem cells originating from various organs [10]–[14]. Unfortunately, the therapeutic benefits of these stem cells were only marginal in most trials. Because the survival and engraftment of donor stem cells after implantation in the damaged heart is essential, the limited benefits of stem cell therapy observed to date might arise from the <5% survival rate of implanted cells 24 hrs after their delivery [15].

Many factors are thought to contribute to the poor survival and engraftment of stem cells after implantation, such as the poor quality of donor stem cells from patients and the unfavorable microenvironment due to inflammation or mechanical stresses [16]–[20]. Using a donor heart model, we have recently demonstrated that the reduction of mechanical stress assists the endogenous regeneration of infarcted hearts by increasing cell proliferation, inhibiting cell apoptosis, and improving stem cell recruitment [16]. However, it is still unknown how mechanical stress affects the exogenous regeneration of injured heart tissue mediated by stem cell therapy.

In this study, we mimicked the dynamic mechanical stresses of a beating heart by using an *in vitro* stretching model that was previously used to induce cardiomyocyte hypertrophy [21], [22]. We applied stretching stimulation to human cardiac stem cells, one of the most promising stem cell sources for heart regeneration, and investigated how the mechanical stresses affected the stem cell's growth, differentiation, and release of paracrine factors.

## Materials and Methods

### Expansion of human cardiac stem cells

The *ex vivo* expansion of human cardiac stem cells was performed as described previously [9], [18], [19], with a few modifications. Briefly, right atrial biopsies (∼100 mg) were obtained from patients who underwent a scheduled open-heart surgery in our department. Biopsies were cut into small pieces, digested with 0.5% trypsin for 5 min, and then cultured as ‘explants’ on dishes coated with fibronectin. After approximately 10 days, we harvested the layer of stromal-like cells surrounding the explants using gentle enzymatic digestion and seeded these cells to form cardiospheres on poly-D-lysine-coated dishes. These cardiospheres were finally reseeded in fibronectin-coated flasks and grown into monolayers for the expansion of cardiosphere-derived cells (CDCs). Twice-passed CDCs were used for the following experiments.

The ethics review board for clinical research at Yamaguchi University approved the protocol (2010025), and the study was conducted in accordance with the Declaration of Helsinki. Written informed consent was obtained from all patients before operation.

### In vitro induction of mechanical stress

**Figure 1:**
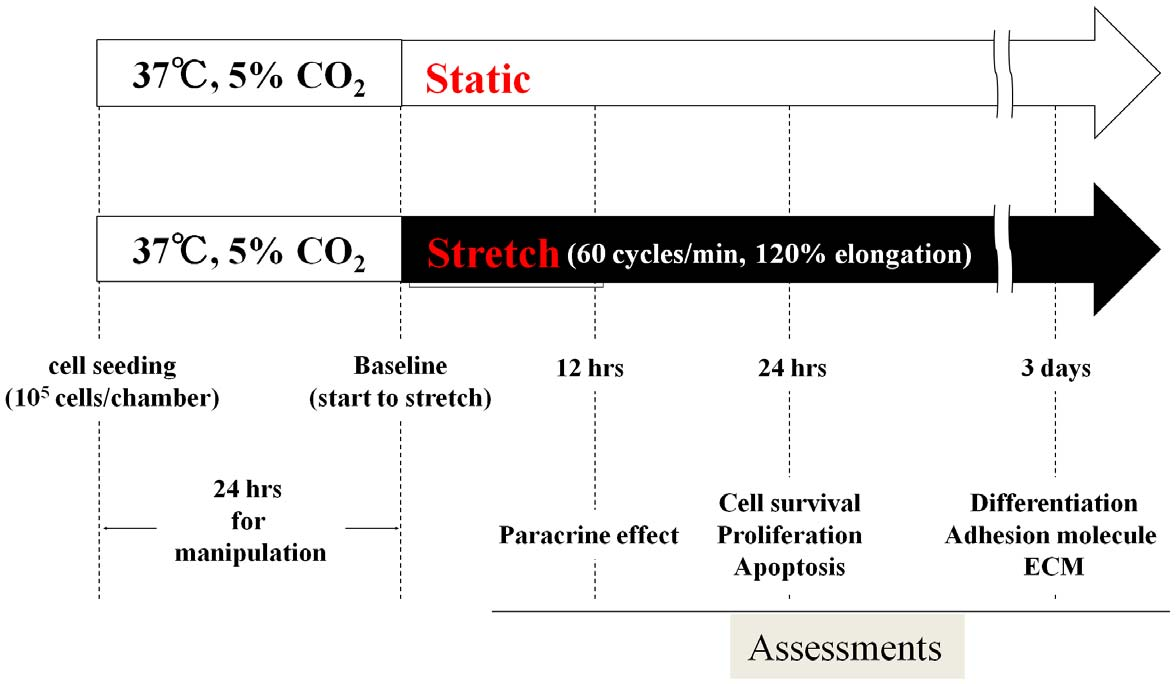
Experimental designs and time schedule of assessments. The cells were seeded at a density of 1×10^5^/chamber. After 24 hrs for manipulation, the chambers were continuously stretched at a frequency of 60 cycles/min with 120% elongation (stretch group). Non-stretched chambers were used as controls (static group). Assessments were performed at the indicated time points.

We applied mechanical stress to CDCs *in vitro* by using a mechanical stretch model (STB-140-10, STREX, Osaka, Japan), which was previously used to induce hypertrophy of cardiomyocytes [21], [22]. The experimental protocol is summarized (***Figure 1***). Briefly, 1×10^5^ cells were seeded in a fibronectin-coated silicon chamber (10 cm^2^, STB-CH-10, STREX, Osaka, Japan). After 24 hrs of incubation for stabilization of cell attachment, mechanical stress was applied to the cells by cyclic stretching of the silicon chamber with 120% elongation in length at a frequency of 60 cycles/min (Stretch group). This stretching stimulation mimics the *in vivo* condition of a beating heart (60 beats/min and 20% fractional shortening). As a control, cells seeded in a fibronectin-coated silicon chamber were cultured without stretching (Static group).

### Cell Growth, proliferation, and apoptosis

Cell growth in both groups was morphologically monitored with phase-contrast microscopy before stretching and after 24 hrs and 3 days of culture. The surviving cells were manually counted under light microscopy (×100) within at least 10 randomly chosen fields.

To further evaluate cell proliferation and apoptosis, cells in both groups were fixed with 1% formaldehyde after 24 hrs of culture. Cell proliferation was identified with fluorescent isothiocyanate (FITC)-conjugated rabbit anti-human Ki67 monoclonal antibody (1∶20 dilution, Abcam). Cells apoptosis was detected with TUNEL staining by using the TMR red in situ cell death detection kit (Roche, Pleasanton, CA) [18], [19]. Cells were also stained with 4′, 6-diamidino-2-phenylindole (DAPI) to visualize the nuclei. Quantitative analyses were performed by counting the positively stained cells under microscopy (×100) within at least 10 randomly chosen fields.

### Scanning electron microscopic preparation

The cultured CDCs were prefixed with 1% glutaraldehyde in 0.1 M phosphate buffer, pH 7.2, (PB) for 1 hour. After washing in PB, they were dehydrated in a graded series of ethanol.All specimens were transferred into t-butyl alcohol. Moreover, they were placed on the specimen stage and frozen by decreasing a stage temperature below −10°C. They were freeze-dried in the apparatus of t-BuOH FREEZE DRYER VFD-21S (Vacuum Device Inc, Ibaragi, Japan) maintaining the stage temperature below 0°C. After complete sublimation of the frozen t-butyl alcohol, the stage temperature was increased up to 25°C, and the dried specimens were then taken out of a main chamber.

The specimens were ion-sputtered with platinum/palladium (10–15 nm) with AUTO FINE COATER JFC-1600 (JEOL Ltd, Tokyo, Japan), and the morphological changes in the CDCs were observed using scanning electron microscopy (SEM) with a Quanta 3D™ microscope (FEI Company, Oregon, USA) at an accelerating voltage of 30 kV.

### Release of cytokines and growth factors

To evaluate how mechanical stress affects the releases of cytokines and growth factors from CDCs, we collected the supernatants from both groups after 12 hrs of culture. The concentrations of various cytokines and growth factors in the supernatants were measured with ELISA kit (R&D Systems, Minneapolis, MN) [17]. We chose to measure VEGF, bFGF, IL-6, IL-1β, SDF-1α, HGF, IGF-1, and TGF-β_1_.

### Expressions of stem cell and differentiation markers

The expression of stem cell and differentiation markers in CDCs in both groups was evaluated with immunostaining after 3 days of culture [18]. Stem cells were identified using PE-conjugated mouse anti-human c-kit monoclonal antibody (1∶20 dilution, eBioscience Inc., San Diego, CA). The expression of marker for cardiomyocytes was identified with rabbit anti-human cardiac troponin I polyclonal antibody (1∶200 dilution, SIGMA-ALDRICH), followed by PE-conjugated goat anti-rabbit second antibody (1∶1000 dilution, R&D systems). The expression of marker for smooth muscle cells was identified with Cy3-conjugated mouse anti-human α-smooth muscle actin (SMA) antibody (1∶200 dilution, SIGMA-ALDRICH). The expression of endothelial marker was identified with PE-conjugated mouse anti-human CD31 monoclonal antibody (1∶100 dilution, eBioscience Inc.). Quantitative analyses involved counting the positively stained cells under microscopy (×100) within at least 10 randomly chosen fields.

### Expression of extracellular matrix and adhesion molecules

To detect whether mechanical stress induces changes in the expression of extracellular matrix (ECM) and adhesion molecules in CDCs from both groups, we performed immunostaining with the purified rabbit anti-focal adhesion kinase (FAK) antibody (1∶25 dilution, SIGMA-ALDRICH) followed by the PE-conjugated goat anti-rabbit polyclonal second antibody (1∶1000 dilution, R&D systems), the purified rabbit anti-integrin-β_1_ (1∶25 dilution, SIGMA-ALDRICH) followed by the PE-conjugated goat anti-rabbit polyclonal second antibody (1∶1000 dilution, R&D systems), the purified rabbit anti-collagen I antibody (1∶25 dilution, Abcam), followed by the Alexa Fluor®-conjugated goat anti-rabbit IgG second antibody (1∶100 dilution, Invitrogen), and the purified rabbit anti-collagen III antibody (1∶25 dilution, Abcam) , followed by the Alexa Fluor®-conjugated goat anti-rabbit IgG second antibody (1∶100 dilution, Invitrogen), after 3 days of culture [18], [23]. Cells were also stained with DAPI, and staining was examined using a fluorescence microscope equipped with a digital camera (Nikon Digital Sight DS-5Mc, JAPAN). The images were semi-quantitatively analyzed using Image-Pro Plus 5.1 (Media Cybernetics, USA) to obtain the intensity of the staining, and the relative intensity of staining was then modified by cell density.

### Statistical analysis

All results are presented as mean ± standard error of the mean. Statistically significant differences between any 2 groups were determined using the paired Student *t* test. Analyses were performed with the use of StatView software (version 5.0), and values of p<0.05 were considered significant.

## Results

### Mechanical stress induced morphological changes and decreased the survival of CDCs

**Figure 2:**
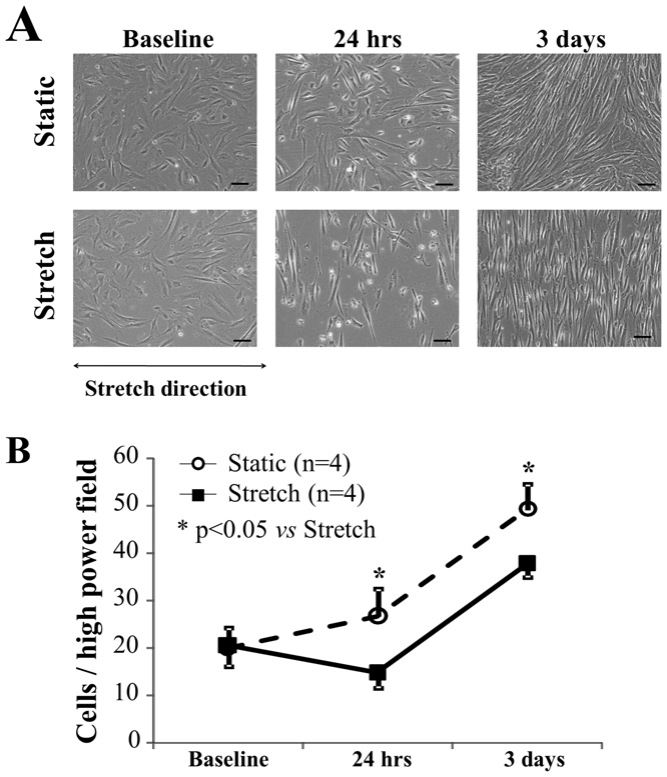
Morphological changes and cell survival. **A**) The cells of both groups showed similar density and morphology at baseline. However, when compared to static culture, stretching stimulation decreased the cell density and caused the cells to be arranged parallel to the stretch direction after 24 hrs and 3 days of culture (bar = 100 µm). **B**) The number of surviving cells was significantly less at 24 hrs and 3 days when the cells were cultured under stretching stimulation compared to under static condition.

**Figure 3:**
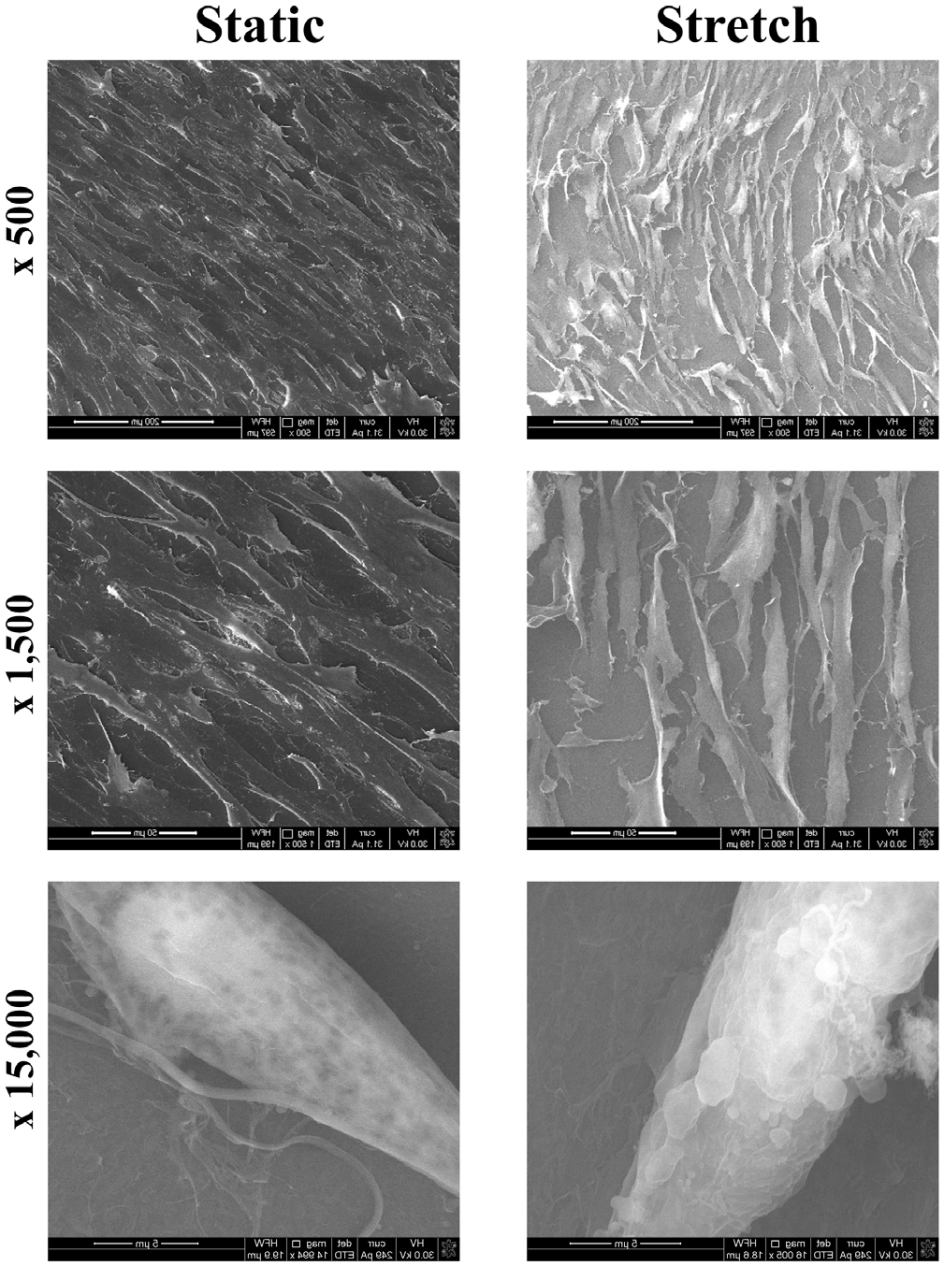
Morphological observation of cells under scanning electron microscopy. Cells in the stretch group were more likely to display a spindle shape, in contrast to the flat shape typically observed in the static group.

After 24 hrs and 3 days of culture, cells were crowded and irregularly oriented in the static group (***Figure 2A***, upper panel). In contrast, the stretch group showed a lower density of cells, and the cells were arranged perpendicular to the stretch direction (***Figure 2A***, lower panel). When observed with scanning electron microscopy, cells from the stretch group were more spindle-shaped, in contrast to the flat shape of the cells from the static group (***Figure 3***).

Quantitative analysis after 24 hrs and 3 days of culture showed that the cell numbers were significantly less in the stretch group than in the static group (p<0.05, ***Figure 2B***), although the cell counts were similar between both groups at baseline.

### Mechanical stress inhibited the proliferation and increased the apoptosis of CDCs

**Figure 4:**
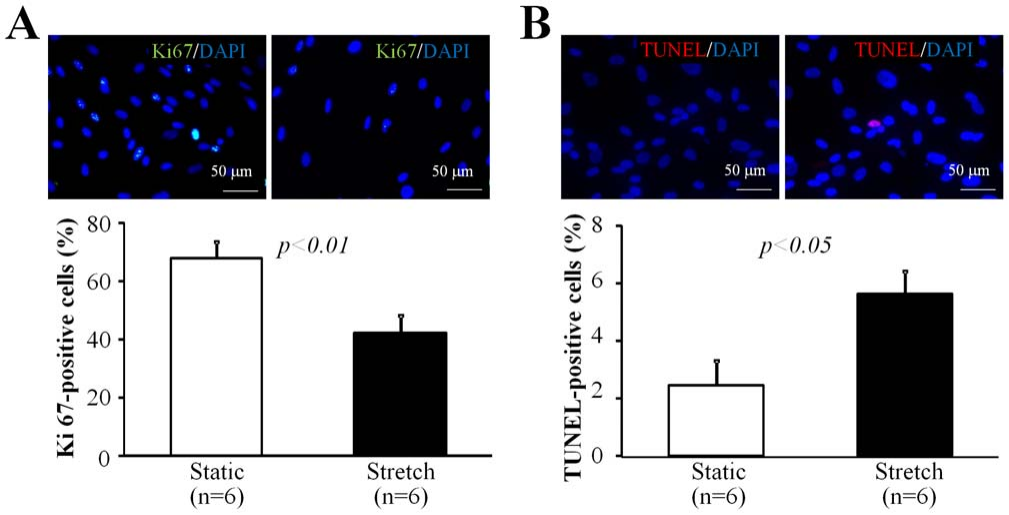
Cell proliferation and apoptosis. The upper panels show representative images of immunostaining for proliferating cells (Ki67-positive, **A**) and apoptotic (TUNEL, **B**) cells. Nuclei were labeled with DAPI. Quantitative analysis showed a significant decrease in the number of proliferative cells (**A**, lower panel) and a significant increase in the number of apoptotic cells (**B**, lower panel) in the stretch group compared with the static group. Data represent 4 separate experiments using different cells.

We measured the proliferation and apoptosis of CDCs by immunostaining analysis after 24 hrs of culture. Compared to the static group, the stretch group showed a significant decrease in Ki-67-positive cells (42.1±6.0% *vs.* 67.7±5.9%, p<0.01; ***Figure 4A***) and a significant increase in TUNEL-positive apoptotic cells (5.6±0.7% *vs.* 2.4±0.8%, p<0.05; ***Figure 4B***).

### Mechanical stress increased the release of inflammatory cytokines and angiogenic factors from CDCs

**Figure 5:**
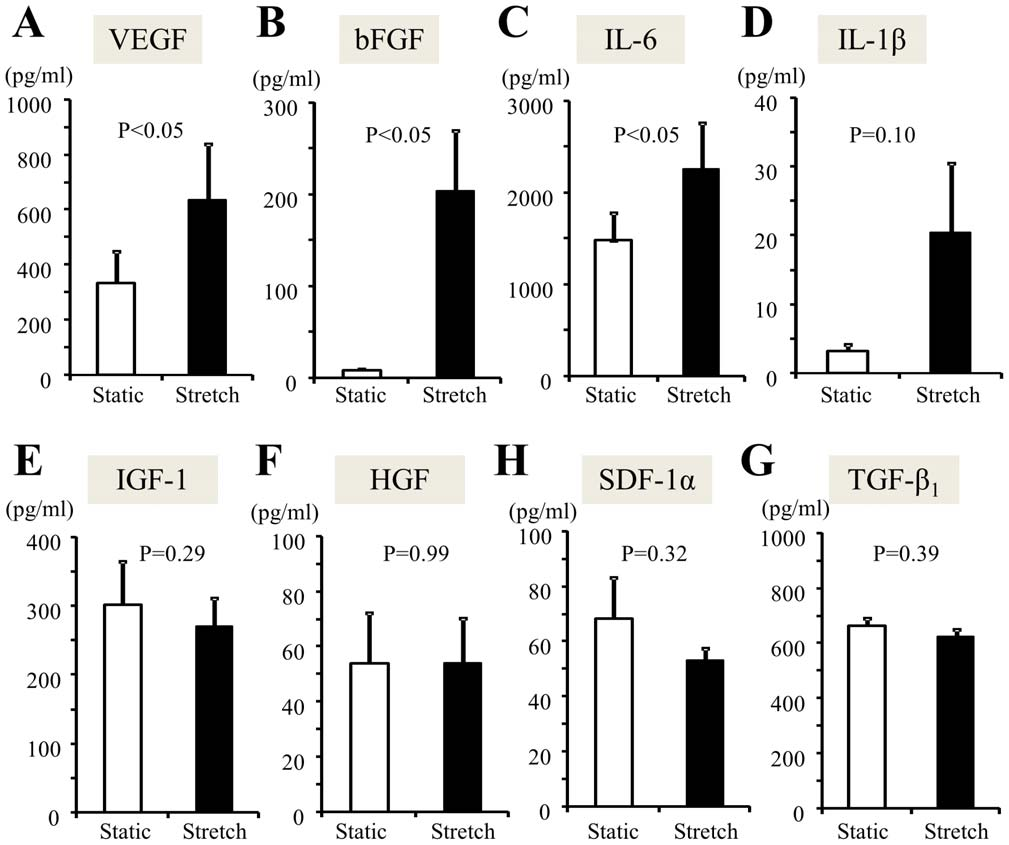
ELISA analysis of cytokines and growth factors released from CDCs. The concentrations of the angiogenic factors, VEGF (**A**) and bFGF (**B**), in conditioned media were significantly higher in the stretch group than the static group. The concentrations of the inflammatory cytokines, IL-6 (**C**) and IL-1β (**D**), were also significantly higher in the stretch group than the static group. In contrast, the concentrations of IGF-1 (**E**), HGF (**F**), SDF-1α (**G**), and TGF-β_1_ (**H**) were not significantly different between the groups. Data represent 6 separate experiments using different cells.

The concentration of angiogenic factors of VEGF and bFGF in the supernatants of the stretch group was approximately 1.9-fold and 29.5-fold higherthan that of the static group, respectively (p<0.05, ***Figure 5A,B***). Mechanical stretching also increased the release of the inflammatory cytokines, IL-6 and IL-1β, from CDCs by approximately 1.5- and 6.3-fold, respectively, compared to the static group (p<0.05, ***Figure 5C,D***).

However, the mechanical stretching stimulation did not significantly change the release of the cytoprotective factors, IGF-1 and HGF (***Figure 5E,F***). Similarly, neither the expression of SDF-1α, a chemokine that is critical for regulating stem cell homing factor, nor TGF-β_1_, a multifunctional growth factor known to accelerate vessel maturation, differed significantly between groups (***Figure 5G,H***).

### Mechanical stress decreased the expression of c-kit, but increased the expression of troponin and SMA

**Figure 6:**
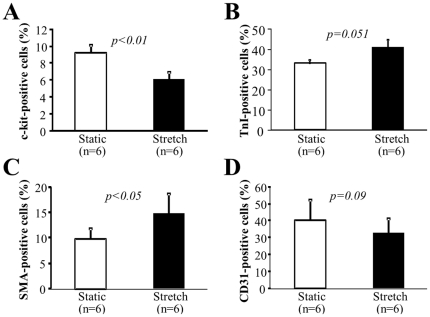
Expression of stem cell and differentiation markers. Immunostaining analyses were used to observe the expression of markers for stem cells (c-kit^+^, **A**), cardiomyocytes (troponin-I^+^ cells, **B**), smooth muscle cells (SMA^+^ cells, **C**), or endothelial cells (CD31^+^ cells, **D**) after 3 days of culture. Representative images of immunostaining are shown in the upper panels. Nuclei were labeled with DAPI. Scale bars represent 100 µm. Quantitative data were derived from six separate experiments using different cells.

The percentage of c-kit-positive cells was significantly higher in the static group compared to the stretch group (9.2±0.8% *vs* 6.0±0.8%, p<0.01, ***Figure 6A***). In contrast, mechanical stretching increased the percentages of cardiac troponin I-positive cells (31.7±2.1% *vs.* 40.6±4.9%, p = 0.051, ***Figure 6B***) and SMA-positive cells (9.9±1.9% *vs.* 14.8±3.9%, p<0.05, ***Figure 6C***). The percentage of CD31-positive cells in the stretch group was slightly lower than in the static group, but this difference was not statistically significant (40.9±11.9% *vs.* 32.6±8.8%, p = 0.09, ***Figure 6D***). These results indicate that mechanical stretching may improve the myogenic differentiations of CDCs.

### Mechanical stress enhanced the expression of adhesion molecules in CDCs

**Figure 7:**
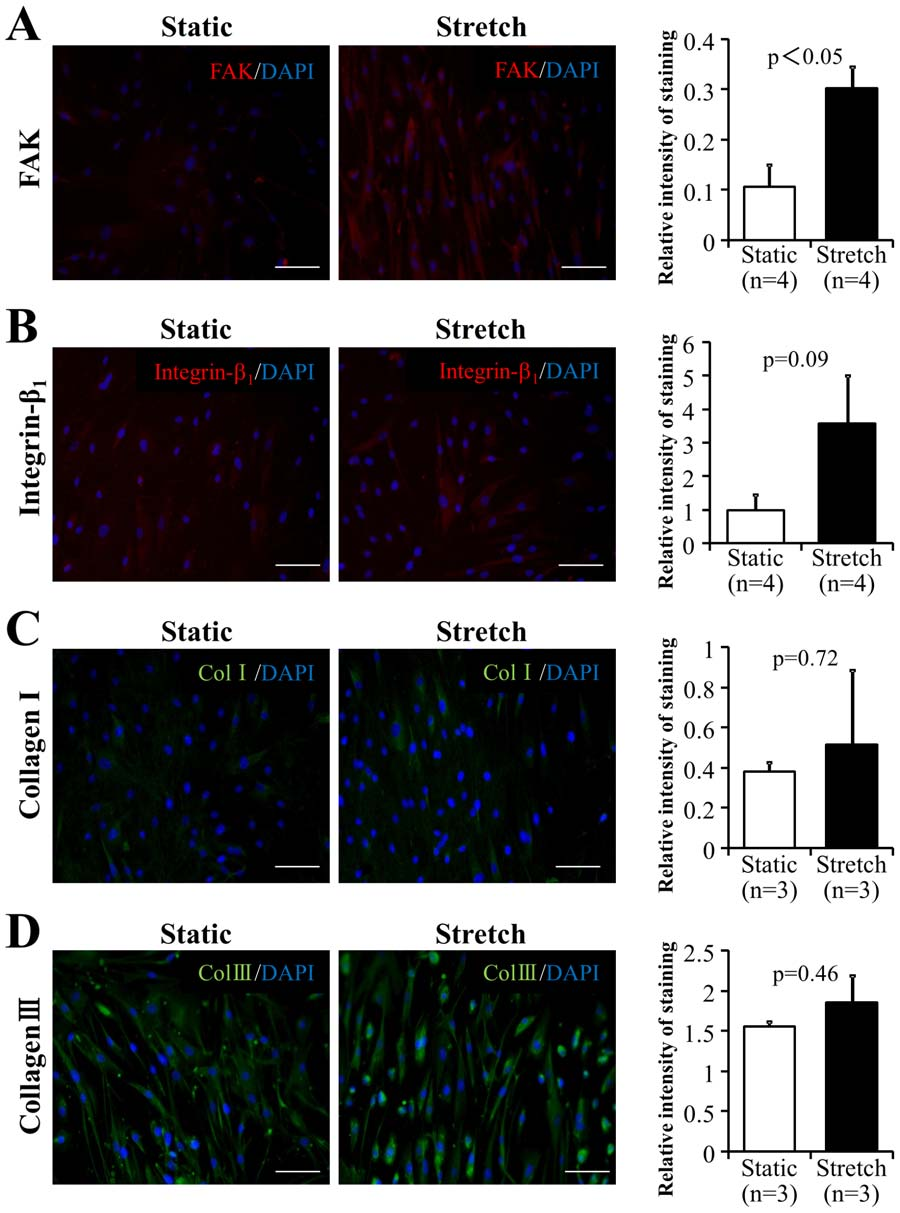
Expression of adhesion molecules and extracellular matrix (ECM). Representative images show the variable expression in stretch and static groups of adhesion molecules and ECM after 3 days of culture with (Stretch) or without (Static) stretching stimulation, as imaged by immunostaining for focal adhesion kinase (**A**), Integrin-β_1_ (**B**), collagen I (**C**), and collagen III (**D**). Nuclei were labeled with DAPI (blue). Semi-quantitative data on the relative intensity of staining was also shown (bar graph on the left). Scale bars represent 100 µm.

Immunostaining analysis of CDCs revealed that the expression of FAK (***Figure 7A***) and integrin-β_1_ (***Figure 7B***), two adhesion molecules that are known to be critical for cell growth and survival, was enhanced by mechanical stretching, although semi-quantitative analysis showed that the expression levels of integrin-β_1_ did not significantly differ between groups due to small sample size. However, the expression of the ECM proteins, collagen I (***Figure 7C***) and collagen III (***Figure 7D***), was not enhanced by mechanical stretching.

## Discussion

By using an *in vitro* stretching model, we clearly demonstrated that mechanical stress affects cardiac stem cells in the following ways: (1) stretch inhibited cell growth by suppressing cell proliferation and increasing cell apoptosis, (2) stretch increased the production of inflammatory cytokines and angiogenic growth factors, (3) stretch improved the expression of markers for cardiomyocytes and smooth muscle cells, and (4) stretch enhanced the expression of adhesion molecules.

Various stem cells or progenitor cells have been clinically used to treat patients with heart failure [10]–[14], [24]. There is increasing evidence that stem cell-based myocardial repair occurs through both direct regeneration and indirect mechanisms (i.e., paracrine effects) [7]–[9]. However, the mechanism by which stem cells repair a damaged heart is still poorly understood because stem cells need to successfully complete a complex set of steps after their delivery into the failing heart [25], including survival, engraftment, the secretion of cytokines and growth factors, differentiation and maturation, and the incorporation with host cells. All these processes are likely affected by the mechanical stress and features of the local tissue microenvironment, such as regional blood flow, extracellular matrix, and cytokines or growth factors. It is essential to define how each of these *in vivo* factors affects stem cell therapy for heart failure.

Cardiac tissue is subjected to dynamic mechanical stress at an early stage of development, and cyclic mechanical stretch plays both physiological and pathological roles in the heart, including the generation and maturation of cardiomyocytes, the regulation of various gap junctions, and the expression of ECM and adhesion molecules [26]–[28]. Because all eukaryotic and prokaryotic cells possess mechanosensitive channels that detect mechanical forces acting on the cell [29], it is possible that mechanical stress impacts the survival and differentiation of donor stem cells after their delivery into a failing heart, which ultimately affects the potency of stem cell therapy *via* direct and/or indirect (paracrine) mechanisms. However, the impact of dynamic mechanical stress on stem cell therapy remains unknown.

In this study, we tried to mimic the dynamic mechanical stress of a beating heart by using an *in vitro* mechanical stretching model that was previously applied to induce cardiomyocyte hypertrophy [21], [22]. This *in vitro* approach enables us to isolate the effects of mechanical stress from other *in vivo* factors that may affect stem cell therapy. We studied CDCs, a heterogeneous population of cells enriched by cardiac stem cells, because (1) the method of *ex vivo* expansion of CDCs from small biopsies is well established [9], [18], [19], (2) CDCs showed a promising angiogenic and cardiogenic potency in several previous studies[9], [15], [18], [19], and (3) clinical trials of CDCs are currently ongoing [24], [25].

Using this *in vitro* approach, we found that mechanical stress stimulation significantly inhibited the growth of CDCs by suppressing cell proliferation and increasing cell apoptosis. Because the survival and engraftment of donor stem cells in the damaged heart was strongly and positively correlated with functional improvement [18], [19], mechanical stress might negate the curative effects of stem cell therapy by inhibiting the survival and growth of stem cells after implantation.

Interestingly, mechanical stress largely enhanced the release of VEGF and bFGF, which are known to stimulate angiogenesis. Similarly, mechanical stress also dramatically increased the release of the inflammatory cytokines, IL-6 and IL-1β. However, mechanical stress did not affect the production of the cytoprotective factors, IGF-1 and HGF. These data suggest that mechanical stress disproportionately changes the production of cytokines and growth factors by stem cells. Transient survivable disruptions of cell plasma membrane integrity are now known to occur in a variety of mechanically active tissues under physiological conditions [30], and contraction-induced cell injury and releases of bFGF and other factors was observed in previous study [31]. It is possible that our stretching model may induce sublethal cell injury and directly release of various growth factors through membrane disruptions. Further experiments, including culturing cardiomyocytes with conditioned medium and then examining the bioactivity and function of cardiomyocytes [8], [26], will be needed to clarify how the complex changes in the paracrine factors affect the curative effects of stem cells therapy.

In a previous study, we demonstrated that a reduction in mechanical stress attenuates the endogenous regenerative potency by increasing the number of c-kit-/Sca-1-positive stem cells in an infarcted heart [16]. The current *ex vivo* results agree well with our previous *in vivo* study because more c-kit-positive cells were counted in CDCs after 3 days of culture in the static group than in the stretch group. Furthermore, mechanical stretching increased the expression of cardiac troponin I and smooth muscle actin in CDCs. These findings suggest that mechanical stress is unfavorable for the growth of cardiac stem cells or progenitor cells but may help to improve their myogenic differentiation and maturation to accelerate the regeneration of new myocardium.

Because ECM and adhesion molecules are critical for the survival and growth of various cells, we also investigated how mechanical stress changed the expression of ECM and adhesion molecules in CDCs. It was not surprising that the expressions of FAK and integrin-β_1_ were observed to be obviously enhanced by mechanical stretching stimulation because mechanical stimulations were previously demonstrated to induce the expression of adhesion molecules in many types of cells [26]–[28]. However, the expression of collagen I and collagen III did not changed significantly by stretching stimulation. The enhanced expression of adhesion molecules suggests the possibility that mechanical stress favors the survival and growth of stem cells after implantation by enhancing the expression of adhesion molecules. Alternatively, it is possible that the enhanced expression of adhesion molecules with mechanical stretching observed in the stretch group of this study may result from a selective survival of those CDCs with an increased expression of adhesion molecules.

The present study was limited to a single condition of cyclic stretching (120% elongation in length at a frequency of 60 cycles/min) and only one type of stem cell (CDCs). Because the response of cells to mechanical stress could be strongly dependent on the conditions of mechanical stress and the type of stem cells, further study is required to confirm our data by using other types of stem cells under different conditions of mechanical stress. However, this uniform *in vitro* mechanical stretch model is not identical to the variable *in vivo* mechanical stress experienced by the cells in a beating heart. Therefore, *in vivo* experiments are ultimately needed to extend the findings of this study.

In conclusion, we developed a simple *in vitro* stretching model that mimics the *in vivo* dynamic mechanical stress of a beating heart. This *in vitro* stretching model can isolate the mechanical stress from other complex factors that change in a failing heart. This was the first time that this *in vitro* model was successfully used to investigate how mechanical stress affects the growth and differentiation of cardiac stem cells and their release of several paracrine factors. The development of these *in vitro* approaches could be helpful for uncovering the complex mechanisms of stem cell therapy.
